# The histone deacetylase Rpd3p is required for transient changes in genomic expression in response to stress

**DOI:** 10.1186/gb-2009-10-5-r57

**Published:** 2009-05-26

**Authors:** Adriana L Alejandro-Osorio, Dana J Huebert, Dominic T Porcaro, Megan E Sonntag, Songdet Nillasithanukroh, Jessica L Will, Audrey P Gasch

**Affiliations:** 1 Department of Biomolecular Chemistry, University of Wisconsin-Madison, University Avenue, Madison, WI 53706, USA; 2 Program in Cellular and Molecular Biology, University of Wisconsin-Madison, Linden Drive, Madison, WI 53706, USA; 3 Laboratory of Genetics, University of Wisconsin-Madison, Henry Mall, Madison, WI 53706, USA; 4 Genome Center of Wisconsin, University of Wisconsin-Madison, Henry Mall, Madison, WI 53706, USA; 5 Current address: Booz Allen Hamilton, Global Health Consulting, Olive Way, Seattle, WA 98101, USA

## Abstract

**Background:**

Yeast responding to stress activate a large gene expression program called the Environmental Stress Response that consists of approximately 600 repressed genes and approximately 300 induced genes. Numerous factors are implicated in regulating subsets of Environmental Stress Response genes; however, a complete picture of Environmental Stress Response regulation remains unclear. We investigated the role of the histone deacetylase Rpd3p, previously linked to the upstream regions of many Environmental Stress Response genes, in producing Environmental Stress Response gene expression changes in response to stress.

**Results:**

We found that the Rpd3-Large complex is required for proper expression of both induced and repressed Environmental Stress Response genes under multiple stress conditions. Cells lacking *RPD3 *or the Rpd3-Large subunit *PHO23 *had a major defect in Environmental Stress Response initiation, particularly during the transient phase of expression immediately after stress exposure. Chromatin-immunoprecipitation showed a direct role for Rpd3-Large at representative genes; however, there were different effects on nucleosome occupancy and histone deacetylation at different promoters. Computational analysis implicated regulators that may act with Rpd3p at Environmental Stress Response genes. We provide genetic and biochemical evidence that Rpd3p is required for binding and action of the stress-activated transcription factor Msn2p, although the contribution of these factors differs for different genes.

**Conclusions:**

Our results implicate Rpd3p as an important co-factor in the Environmental Stress Response regulatory network, and suggest the importance of histone modification in producing transient changes in gene expression triggered by stress.

## Abstract 2

Chromatin-immunoprecipitation and computational analysis implicate Rpd3p as an important co-factor in the network of genes regulating the yeast environmental stress response.

## Background

Sudden environmental changes can trigger rapid and dramatic changes in genomic expression. This involves coordinated expression of hundreds to thousands of genes, whose expression is precisely modulated in timing and magnitude. Many different transcription factors function in the cell at any given time and respond to distinct upstream signals. Therefore, cells must integrate the action of numerous signals and regulatory factors to produce a coherent genomic expression program customized for each new environment.

Yeast respond to stress in part by initiating the Environmental Stress Response (ESR), which consists of approximately 600 genes whose expression is repressed and approximately 300 genes whose expression is induced by diverse stresses [1,2]. The repressed genes include approximately 130 ribosomal protein ('RP') genes and a distinct group of approximately 450 genes more broadly related to protein synthesis ('PS genes'). Both groups are highly expressed in actively growing cells but sharply repressed, with slightly different expression profiles, in response to stress. Genes induced in the ESR ('iESR genes') are involved in varied aspects of stress defense, including redox regulation, protein folding, osmo-tolerance, cell signaling, and other functions. Initiation of the ESR is not required to survive the offending stress but rather helps to protect cells against subsequent severe doses of the same or different stress (although it cannot fully explain acquired stress resistance in all cases) [3].

Although activated by many different stresses, the ESR is regulated differently depending on the environment. Numerous upstream signaling pathways have been implicated in condition-specific ESR regulation, including the high osmolarity glycerol (HOG) [4] (Jessica Clarke and APG, unpublished data), MEC [5], and protein kinase C (Scott Topper and APG, unpublished) pathways in response to osmotic shock, DNA damage, or reducing agents, respectively, and the protein kinase A and target of rapamycin (TOR) pathways upon stress relief [6-10] (reviewed in [11]). Furthermore, different subsets of iESR genes can be induced by stress-specific transcription factors, such as the oxidative-stress factor Yap1p [1], the heat shock factor Hsf1p [12-14], Sko1p and Hot1p upon osmotic stress [15-18], and the 'general-stress' transcription factors Msn2p and Msn4p in response to diverse stresses (reviewed in [11]). However, little is known about how these signals are integrated to mediate ESR initiation, or how genes repressed in the ESR are coordinated with genes induced in the program.

One mechanism of altering gene expression is through changes in chromatin state. The histone deacetylase Rpd3p deacetylates histones in both coding and noncoding regions, where it is thought to function in at least two distinct complexes (reviewed in [19,20]). A small complex (Rpd3S) suppresses cryptic transcription initiation by deacetylating histones after elongating polymerase [21-23]. Rpd3S is recruited via the combined action of the Eaf3p and Rco1p subunits to histone H3 methylated by Set2p during transcription of the open reading frame [21-23]. In contrast, a large complex (Rpd3L) is recruited to promoters by site-specific DNA binding proteins, including the Ume6p subunit of Rpd3L, where it is thought to function in transcription initiation [23-27]. Rpd3p is known to bind different promoters under different conditions, such as cold shock and rapamycin treatment [28-30]. In fact, many promoters to which Rpd3p relocalizes are of genes repressed in the ESR. The effects of Rpd3p at these promoters have not been shown on a global scale, but the result suggests Rpd3p is required for stress-dependent repression of ESR genes [11,30].

Although traditionally linked to repression, histone deacetylases can also function during gene activation [31-36]. Induction of several different yeast genes requires Rpd3p following salt treatment, hypoxia, or DNA damage [32-34]. The precise mechanism is not clear but requires Rpd3p for recruitment of RNA polymerase to promoters of genes (including iESR genes) induced by osmotic shock and DNA damage [32,34]. Furthermore, induction of hypoxic genes requires Rpd3p-dependent histone deacetylation for nucleosome displacement and stable binding of the Upc2p transcription factor within the genes' regulatory regions [33]. That Rpd3p has been linked to stress-dependent gene induction and repression raised the possibility that Rpd3p participates in regulating both induced and repressed genes within the ESR.

Indeed, here we show that Rpd3p is required for proper initiation of the ESR, including normal regulation of both induced and repressed genes, in yeast responding to multiple stresses. Cells lacking *RPD3 *or the Rpd3L subunit *PHO23 *had a major defect, specifically during the transient phase immediately after H_2_O_2 _treatment, while cells lacking the Rpd3S subunit *RCO1 *did not. Chromatin-immunoprecipitation (ChIP) at candidate ESR genes revealed that Rpd3p moves to numerous promoters upon stress to mediate histone deacetylation; however, the precise pattern of chromatin change was different for different nucleosomes and genes investigated. We show that Rpd3p binds directly to genes induced by stress and is required for normal binding of Msn2p to numerous promoters. Together, this work implicates Rpd3L as an important co-factor in the ESR regulatory network.

## Results

### Rpd3p is required for the full dynamic range of stress-activated gene expression changes

**Figure 1:**
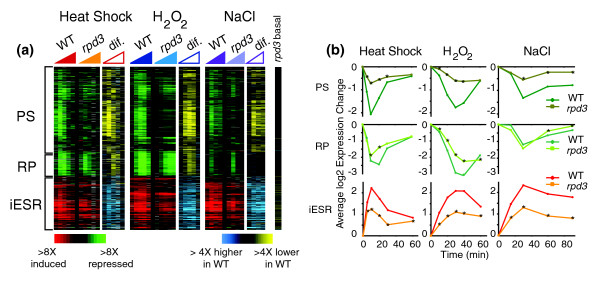
Rpd3p is required for stress-dependent activation of the environmental stress response. Gene expression in wild-type and *rpd3Δ *cells responding to 25°C to 37°C heat shock (left panels), 0.4 mM H_2_O_2 _treatment (middle panels), or 0.75 M NaCl exposure (right panels) as described in Materials and methods. **(a) **The gene expression diagram represents the induced (red) or repressed (green) expression measurements of each gene (represented as rows) in the protein synthesis (PS), ribosomal protein (RP), and induced environmental stress response (iESR) gene groups for each microarray experiment (represented as columns organized temporally within each time course). The difference ('dif.') between wild type and *rpd3Δ *is represented to the right of each expression diagram: yellow indicates weaker repression and blue indicates weaker induction in the *rpd3Δ *mutant. Basal expression differences between *rpd3Δ *and wild type grown in the absence of stress are also shown. **(b) **The average log_2 _expression change of genes in the PS, RP, and iESR subgroups shown in (a) plotted for wild type and *rpd3Δ *cells. Time points with statistically smaller changes in expression in *rpd3Δ *cells (*P *< 0.01, paired *t*-test) are indicated with an asterisk.

We followed genomic expression in wild-type and *rpd3Δ *cells responding over time to a 25°C to 37°C heat shock, 0.4 mM H_2_O_2_, and 0.75 M NaCl. A large fraction (56 to 80%) of the gene expression changes seen in wild-type cells was affected by *RPD3 *deletion, and this included both repressed and induced genes (Table 1). In particular, Rpd3p was required for normal expression of the vast majority of ESR genes (Figure 1). Repression of PS genes was heavily dependent on Rpd3p in response to all stresses, whereas repression of RP genes required Rpd3p for full repression in response to heat and H_2_O_2 _stress but not salt treatment. Normal induction of iESR genes also required Rpd3p, since the *rpd3Δ *strain displayed more than twofold decreased induction levels at the peak of the response. Interestingly, a subset of iESR genes (approximately 50% at a false discovery rate of 0.05) showed slight derepression (approximately 1.5-fold) in the *rpd3Δ *mutant in the absence of stress (Figure 1; Figure S1 in Additional data file 1). The defect in stress-dependent induction was not due to an already activated stress response in mutant cells, indicated by normal cytosolic localization of Msn2p before stress but substantial Msn2p nuclear accumulation after stress, similar to wild-type (Figure S2 in Additional data file 1). Furthermore, these iESR genes (as well as those with no significant difference in basal expression) still had a defect in induction beyond what could be accounted for by basal expression differences (Figure S1 in Additional data file 1). Thus, Rpd3p is required for the induction and repression of ESR genes during stress, although each ESR subgroup shows a qualitatively different dependence on the protein.

**Table 1:** Genes affected by *RPD3 *deletion

	Heat shock	H_2_O_2_	NaCl	Common*
Wild type^†^	2,089	2,082	2,421	996
Rpd3p-affected^‡^	1,643 (79%)	1,175 (56%)	1,696 (70%)	562 (56%)

*Genes common to all three stresses. ^†^Genes affected in wild-type cells (see Materials and methods). ^‡^Genes whose expression change was defective in the *rpd3Δ *strain relative to wild type, based on time-course analysis (see Materials and methods).

Stress-dependent gene expression changes are often transient, in that large changes immediately after stress subsequently relax to new 'steady-state' levels as cells acclimate (reviewed in [37]). We found that Rpd3p is particularly important for this transient phase of expression (Figure 1b). PS genes showed almost no transient expression changes, while iESR genes showed reduced expression levels specifically at the peak of the transient phase. RP genes also showed diminished expression differences at the peak of the response to heat shock and H_2_O_2 _treatment. Despite the defect in transient ESR expression, the *rpd3Δ *mutant eventually reached near-wild-type expression changes by the end of these time courses. This indicates that Rpd3p is not necessarily required to maintain new steady-state levels of expression in cells acclimated to high temperature or H_2_O_2_, but is critical in producing a large, rapid response to stress.

### ESR regulation requires histone deacetylase activity through the Rpd3L complex

We found that the catalytic activity of Rpd3p, as well as modifiable histones and subunits of the Rpd3L complex, were required for proper ESR regulation. Cells harboring the catalytically inactive *rpd3-H150:151A *protein [32] or treated with the Rpd3p inhibitor trichostatin A displayed the same widespread defect as the *rpd3Δ *strain (Figure S3 in Additional data file 1). A similar defect was observed in cells harboring a mutant histone H4 (H4KQ), in which amino-terminal lysines were changed to glutamine to mimic the acetylated histone state [38] (Figure S3 in Additional data file 1). This effect was particularly clear for PS and iESR genes, although there was only a subtle defect in repression of the RP genes in the H4 mutant strain.

To distinguish between the effects of the different Rpd3p complexes, we characterized the H_2_O_2 _response in cells lacking Pho23p or Rco1p, exclusive members of the Rpd3L and Rpd3S complexes, respectively [21,23,39]. The expression defect seen in the *pho23Δ *mutant, but not the *rco1Δ *cells, was highly similar to that in the *rpd3Δ *mutant. Over 80% of Rpd3p-affected genes were equally dependent on Pho23p (R = 0.94, m = 0.98), whereas less than 12% of Rpd3-affected genes showed a partial expression defect in cells lacking *RCO1*. Furthermore, the *pho23Δ *strain showed the same defect in transient expression as the *rpd3Δ *cells (Figure S4 in Additional data file 1). In contrast, the *rco1Δ *cells showed large changes in expression similar to wild type, albeit with a slightly delayed response that is difficult to interpret due to spurious internal transcripts in this mutant [21,23]. Nonetheless, these data show that defects in the magnitude and transience of gene expression can be accounted for by the Rpd3L complex. Consistent with previous studies [28,40,41], we found few of the Rpd3L-dependent expression changes were dependent on the Ume6p subunit (data not shown), which is thought to recruit the complex to specific loci [24,25,27]. This suggests that other DNA binding proteins may be required for Rpd3L-dependent gene expression changes (see below).

### Representative ESR genes show Rpd3p-dependent changes in chromatin following stress

**Figure 2:**
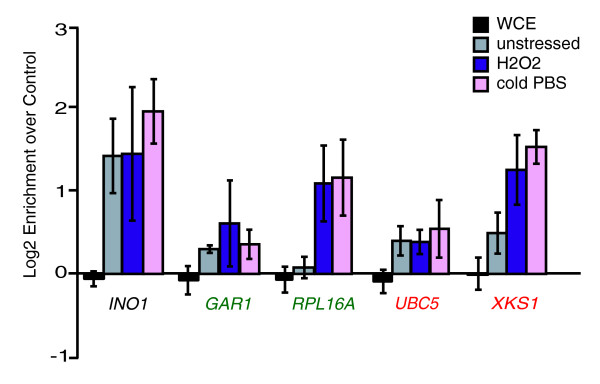
Rpd3p is bound upstream of several target genes after stress. Rpd3-myc binding upstream of several genes (including the positive control *INO1*, PS gene *GAR1*, RP gene *RPL16A*, and iESR genes *UBC5 *and *XKS1*) was assessed using ChIP before and 10 minutes after 0.4 mM H_2_O_2 _treatment or cold phosphate-buffered saline shock (see Materials and methods for details). The log2 enrichment of each fragment recovered from the Rpd3-myc expressing strain versus an untagged control strain is shown, for unstressed cells and cells responding to stress, according to the key on the right. Error bars represent the standard deviation of biological triplicates. The enrichment of each locus in whole-cell extracts (WCE) is shown as a control.

Previous studies showed Rpd3p physically bound to many ESR-gene promoters during times of stress [28-30]. Global studies probing Rpd3p binding after cold shock (inadvertently inflicted by [28]) and rapamycin treatment [29] showed that promoters of 60% of PS genes (*P *< 10^-32^) and 90% of RP genes (*P *< 10^-20^) were bound by Rpd3p. Few of these regions are bound under standard conditions [29,30]. Roughly 20% of iESR-gene upstream regions were bound by Rpd3p under stress conditions, though this may be an underestimate, since chromatin-remodeling enzymes are difficult to ChIP, particularly during dynamic responses [28]. Consistent with these studies, we found Rpd3p bound upstream of four representative ESR genes (including one PS, one RP, and two iESR genes) after H_2_O_2 _treatment (Figure 2). Three of the targets also showed some Rpd3p binding before stress, and all but the *UBC5 *promoter showed increased Rpd3p binding after H_2_O_2 _treatment. These results were similar to those seen in cold-shock (Figure 2), suggesting that many of the previously observed binding events from [28] also occur during H_2_O_2_ stress.

**Figure 3:**
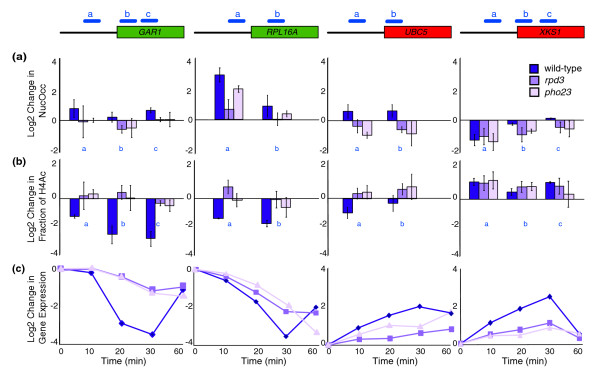
Rpd3p mediates stress-dependent changes in histone acetylation. Changes in nucleosome occupancy (NucOcc) and histone H4 acetylation (H4Ac) at specific nucleosomes (blue bars) spanning representative repressed (green) and induced (red) ESR genes shown in Figure 2 was measured in wild-type, *rpd3Δ *and *pho23Δ *cells responding to 0.4 mM H_2_O_2 _treatment (see Materials and methods for details). The log2 changes in **(a) **nucleosome occupancy and **(b) **fraction of nucleosomes acetylated on H4 following H_2_O_2 _exposure is shown for each gene. Error bars represent the range of two replicates for wild type or the standard deviation of at least three experiments for *rpd3Δ *and *pho23Δ*. H4 acetylation levels were normalized to levels of nucleosome occupancy to capture the change in the fraction of acetylated nucleosomes. **(c) **Expression changes of each gene as measured by microarray experiments at 10, 20, 30, 40, and 60 minutes after H_2_O_2 _treatment in wild-type, *rpd3Δ *and *pho23Δ *cells, according to the key shown.

We therefore characterized changes in nucleosome occupancy and H4 acetylation at nucleosomes spanning the same four ESR genes in wild-type, *rpd3Δ *or *pho23Δ *strains using mononucleosome digestion and ChIP of acetylated H4 before and after H_2_O_2 _exposure. The results showed different trends at different genes. Nucleosomes at repressed ESR genes *GAR1 *and *RPL16A *showed Rpd3L-dependent changes in histone deacetylation following H_2_O_2 _treatment. Though wild-type cells showed an approximately three- to eightfold decrease (depending on the gene and nucleosome) in the fraction of acetylated nucleosomes (Figure 3b), both the *rpd3Δ *and *pho23Δ *mutants had a major defect in histone deacetylation across both repressed ESR genes. This defect correlated with the defect in their H_2_O_2_-dependent repression (Figure 3c). Interestingly, the *rpd3Δ *mutant, and to some extent the *pho23Δ *strain, also had a defect in nucleosome repositioning at these repressed genes: whereas wild-type cells responding to H_2_O_2 _showed a dramatic increase in nucleosome occupancy upstream of *RPL16A*, the *rpd3Δ *mutant showed a major defect in this response (Figure 3a). The *pho23Δ *mutant displayed a weaker defect than the *rpd3Δ *strain, indicating that Pho23p is only partially required for the stress-dependent increase in nucleosome occupancy at this locus. Together with results in Figure 2, this indicates that Rpd3L-dependent histone deacetylation is required for repression of these PS and RP genes.

The two representative iESR genes each displayed a unique profile in chromatin change. Nucleosomes surrounding the transcription start site of the induced gene *UBC5 *displayed decreased histone acetylation in wild-type cells but not the *rpd3Δ *or *pho23Δ *mutants responding to H_2_O_2_. In addition, nucleosome occupancy at these loci increased in wild-type cells, but not the mutants. In contrast, both the promoter and open reading frame of iESR gene *XKS1 *showed increased histone acetylation and nucleosome loss in wild-type cells, with no significant defect in either mutant. Nonetheless, this gene showed approximately threefold weaker induction in the *rpd3Δ *and *pho23Δ *mutants, specifically during the transient phase of expression. This reveals a decoupling of chromatin changes upstream of *XKS1 *and *XKS1 *gene induction in the mutant strains responding to stress, in a manner dependent on direct Rpd3p binding to the region (see Discussion).

### Implication of Rpd3p-dependent and -independent transcriptional regulators

The above results indicate that Rpd3p has different effects at different ESR genes, perhaps due to different regulators functioning at those genes. To identify additional stress-dependent regulators, we systematically analyzed clustered expression data for enrichment of known transcription factor targets or functional gene groups. We manually identified gene clusters in the hierarchically clustered dataset and scored enrichment of Gene Ontology annotations [42], targets of known transcription factors [43], and genes with different upstream *cis*-regulatory elements [44]. This analysis pointed to transcription factors involved in the Rpd3p-dependent and Rpd3p-independent regulation of gene expression (Table S1 in Additional data file 2).

Multiple clusters of Rpd3p-dependent induced genes were enriched for genes with upstream Msn2p and Msn4p binding sites (CCCCT [45,46]), consistent with the known role of Msn2/4p in regulating iESR genes [1,46,47]. Another cluster of Rpd3-dependent repressed genes was heavily enriched for genes with upstream Polymerase A and C (PAC; GCGATGAG) elements and Ribosomal RNA Processing Elements (RRPEs; AAAAWTTTT), known to be enriched in PS genes and previously linked to promoters bound by Rpd3p [1,28,41,48]. Another cluster was enriched for proteasome genes and genes containing binding sites of the proteasome regulator Rpn4p. These associations raise the possibility that Rpd3p may work with these factors to mediate the observed gene expression changes (see more below).

**Figure 4:**
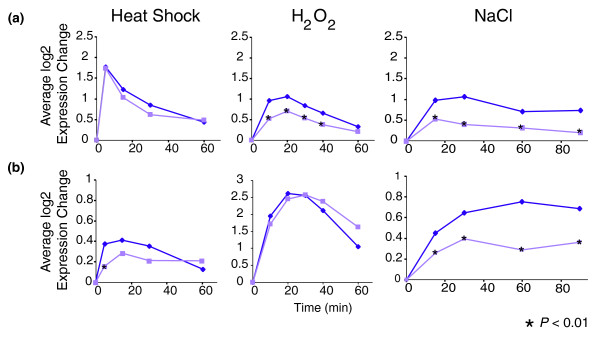
Targets of Hsf1p and Yap1p show conditional dependence on Rpd3p. The average expression of **(a) **Hsf1p targets [14] or **(b) **Yap1p targets [1] was plotted for wild-type (dark purple) and *rpd3 *(light purple) cells responding to heat shock (left panels), H_2_O_2 _treatment (middle panels), or NaCl exposure (right panels) as described in Materials and methods. Time points with smaller expression changes in *rpd3Δ *cells (*P *< 0.01, paired *t*-test) are indicated with an asterisk.

Interestingly, we identified some genes whose expression was conditionally dependent on Rpd3p. Targets of the heat shock transcription factor Hsf1p or the oxidative stress transcription factor Yap1p were only dependent on Rpd3p in response to specific conditions (Figure 4). The majority of Hsf1p targets did not require Rpd3p for induction following heat shock but showed Rpd3-dependent induction in response to H_2_O_2 _and NaCl treatment (Figure 4a). Similarly, induction of Yap1p targets (Figure 4b) was independent of Rpd3p in response to H_2_O_2_, while a subset induced with the ESR required Rpd3p for full induction following heat shock and salt stress only. Hsf1p and Yap1p are known to be condition-specific regulators of subsets of iESR genes, functioning during heat shock and oxidative stress, respectively (reviewed in [11]). Under other conditions, many of these genes are regulated by Msn2/4p. Our observations are consistent with the model that Hsf1p and Yap1p function independently of Rpd3p to regulate gene induction, whereas Msn2/4p act in an Rpd3p-dependent manner.

### Rpd3p is required for normal Msn2p binding and transcription initiation

**Figure 5:**
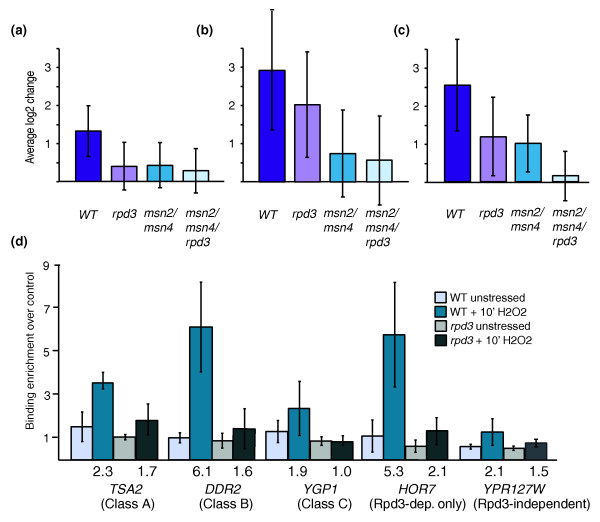
Rpd3p is required for proper Msn2/4p action. **(a-c) **Gene expression measured in wild-type (WT), *rpd3Δ*, *msn2Δ */*msn4Δ*, and *msn2Δ */*msn4Δ */*rpd3Δ *cells treated with 0.4 mM H_2_O_2 _for 30 minutes. Average log_2 _expression changes of (a) 215 genes equally affected by deletion of *RPD3*, *MSN2*/*MSN4*, or *MSN2*/*MSN4*/*RPD3*, (b) 83 genes affected more by deletion of *MSN2*/*MSN4 *than *RPD3*, and (c) 103 genes that display additive dependence on *RPD3 *and *MSN2*/*MSN4*. The standard deviation of the genes' expression is shown for each gene group. **(d) **Msn2p binding before and 10 minutes after 0.4 mM H_2_O_2 _treatment in wild-type and *rpd3Δ *cells, according to the key for: *TSA2 *(from (a)), *DDR2 *(from (b)), *YGP1 *(from (c)), *HOR7 *(dependent on Rpd3p only), and *YPS127W *(dependent on Msn2/4p but not Rpd3p). Fold-change in Msn2p occupancy between stressed and unstressed cells is listed below each plot. Error bars represent the standard deviation of triplicate experiments.

To investigate the link between Msn2/4p and Rpd3p function, we measured genomic expression in strains lacking *RPD3*, *MSN2*/*MSN4*, or *MSN2*/*MSN4*/*RPD3 *as cells responded to H_2_O_2_. Interestingly, genes fell into different categories depending on their expression defect (Figure 5). One class of genes was equally dependent on Rpd3p and Msn2/4p for induction, with no additional defect in the triple mutant (Figure 5a). A second class required both sets of factors but was more dependent on Msn2/4p (Figure 5b), while a third class suggests redundant function of Rpd3p and Msn2/4p at these genes (Figure 5c). The latter group was enriched for genes involved in carbohydrate metabolism (*P *< 10^-8^) and trehalose synthesis (*P *< 10^-5^), suggesting functional relevance of the categorization. A fourth class of genes was dependent only on Rpd3p (data not shown), indicating that additional Rpd3p-dependent transcription factors are required for proper initiation of the ESR (including Rpn4p and others). Importantly, a fifth group of genes was dependent only on Msn2/Msn4p (data not shown), which underscores that the Rpd3p-dependent defect in iESR-gene induction is not simply caused by failure to activate Msn2/4p, consistent with microscopy data (Figure S2 in Additional data file 1). Thus, most but not all of Msn2/4p-dependent genes require Rpd3p for full induction, and these targets show qualitative differences in their dependence.

These results suggest Rpd3p may be required for Msn2/4p action during gene induction. We therefore measured Msn2p binding upstream of genes representing each category above in wild-type and *rpd3Δ *cells responding to H_2_O_2 _(Figure 5d). While none of the promoters tested showed Msn2p bound before stress, as expected, wild-type cells showed an increase in Msn2p promoter binding that was defective in the *rpd3Δ *strain at most targets, regardless of class. The exception was *YPR127W*, an Msn2p-dependent but Rpd3p-independent target, which showed no significant defect in Msn2p binding in the *rpd3Δ *strain. Thus, Rpd3p was required for Msn2p binding upstream of targets that showed dependence on Rpd3p for induction.

It is important to note that over half the H_2_O_2_-induced gene expression changes were not affected by *RPD3 *deletion or *MSN2*/*4 *deletion. This underscores that Rpd3p is not universally required for all gene expression changes in response to stress, and shows that the defect in expression is not due to a gross alteration in the *rpd3Δ *mutant's response.

### Rpd3p is required for ESR suppression following stress relief

That Rpd3p is implicated in both gene induction and repression following stress treatment raised the possibility that Rpd3p participates in the reciprocal regulation of the same genes during stress relief, when the ESR is suppressed. To test the role of Rpd3p in ESR suppression, we measured gene expression in wild-type and *rpd3Δ *cells acclimated to 37°C as cells were returned to 25°C. Strikingly, the *rpd3Δ *strain had a significant defect in ESR suppression during stress relief (Figure S5 in Additional data file 1): whereas wild-type cells rapidly repressed expression of iESR genes in response to stress relief, *rpd3Δ *cells displayed a significantly weaker response. Similarly, induction levels of PS genes were significantly smaller in the *rpd3Δ *strain compared to the wild-type cells recovering from stress. Consistent with results presented above, the RP genes were distinct in that induction upon stress relief was only mildly affected by *RPD3 *deletion. These results suggest that Rpd3p is not exclusively required for the repression or for the induction of the ESR genes but instead is required for proper changes in the genes' expression regardless of the directionality of the change.

## Discussion

Our results reveal that Rpd3p is required for many stress-dependent gene expression changes, particularly genes in the yeast ESR. We show that Rpd3p and the Rpd3L subunit Pho23p (but not the Rpd3S component Rco1p), as well as Rpd3p catalytic activity and modifiable histones, are required to produce these effects. Rpd3p binds directly to promoters of representative ESR genes, indicating that the Rpd3-dependent changes in chromatin structure that we see are direct at these promoters. Furthermore, the observed defects in iESR induction correlate with decreased Msn2p binding at candidate promoters in the *rpd3Δ *strain. Together with previous global studies of Rpd3p localization [28-30], these results indicate that Rpd3L acts directly at many ESR genes to mediate transient changes in gene expression. The defect in stress-activated expression leads to a corresponding defect in acquired stress resistance (Figure S6 in Additional data file 1), similar to that we have previously shown in cells lacking Msn2p and/or Msn4p [3]. Thus, Rpd3p is an important cofactor in initiating the ESR. Models for how Rpd3p fits into the ESR regulatory network are discussed below.

### Role of Rpd3p in ESR initiation under diverse stress conditions

Rpd3p likely acts with distinct transcription factors at different classes of ESR promoters. PS genes are heavily enriched for upstream PAC elements (GCGATGAG) and RRPEs (AAAAWTTTT) [1,48], which have also been linked to Rpd3p binding [28]. Recently, the binding proteins of both elements have been identified and linked to PS expression. PAC is bound by Dot6p and Pbs1p [49,50], and deletion of the two genes leads to defective PS gene repression in response to heat shock [50]. The RRPE binding factor was recently identified as Stb3p, which interacts with the Sin3p subunit of Rpd3p complexes [51,52] and is required for PS gene induction upon starvation relief but represses PS gene transcription when overexpressed (D Liko and W Heideman, personal communication). Although we found no expression defect in an *stb3Δ *mutant responding to stress (data not shown), the link between Stb3p, Sin3p/Rpd3p, and RRPEs suggests that the proteins function together at this regulatory motif to affect PS gene expression.

Rpd3p has a distinct role in repressing RP genes, since their expression was mildly Rpd3L-dependent under certain conditions only. Nonetheless, we found that Rpd3p moves to the promoter of *RPL16A *upon H_2_O_2 _treatment (Figure 2), as previously found in response to cold shock [28,30], and is required for normal histone deacetylation and nucleosome deposition/repositioning (Figure 3). Rpd3p has previously been linked to RP gene repression after rapamycin treatment [29,53,54], although we found no requirement for the proposed repressor Crf1p (data not shown) [55]. We have, however, found a requirement for the ATP-dependent nucleosome-remodeling complex, RSC, which is important for proper nucleosome organization upstream of many genes [49]. RSC mutants have increased RP expression in the absence of stress [56], while cells lacking Rsc1p fail to fully repress RP expression and, to some extent, PS gene expression upon H_2_O_2 _treatment (our unpublished data). Like Rpd3p, RSC binds RP promoters in a condition-specific manner [57]. Thus, Rpd3p and RSC may function in parallel pathways at these genes. Interestingly, stress-dependent changes in nucleosome occupancy at *RPL16A *were only partially dependent on Pho23p, raising the possibility that Rpd3L functions partially independently of Pho23p or that Rpd3p is acting through multiple complexes, at least one of which does not require Pho23p [20-23].

The role of Rpd3L at iESR genes is less clear; however, our ChIP experiments suggest four general models for how Rpd3p may affect gene induction. The first is that some iESR genes may be indirectly affected by Rpd3L activity, particularly those for which there is no evidence of Rpd3p binding in response to stress. The second model is that Rpd3p plays an important and direct role in repressing iESR expression in the absence of stress, since Rpd3p binds directly to the promoters of *UBC5 *and *XKS1 *before stress (Figure 2) and these genes (plus nearly half of iESR genes) show slight derepression under normal conditions (Figure 1). This model is not incompatible with separate roles for Rpd3p in regulating stress-dependent expression changes, demonstrated by *UBC5 *and *XKS1*. At the *UBC5 *promoter, Rpd3p directly deacetylates promoter-based histones to mediate gene induction. This is consistent with results of De Nadal *et al*. [32], who showed Rpd3-dependent histone deacetylation is required for polymerase recruitment. In contrast, H_2_O_2_-dependent chromatin changes at *XKS1 *were not detectibly dependent on Rpd3L, despite increased Rpd3p binding upon treatment. The *rpd3Δ *mutant ultimately induced *XKS1 *to levels higher than wild type, but with a major defect in the normal transient burst of expression. Thus, the changes in histone acetylation did not lead to normal gene induction. One possibility is that gene induction triggered by H_2_O_2 _requires proper Rpd3-dependent promoter architecture before stress; alternatively, Rpd3p may play a role late in gene induction, after active nucleosome acetylation, as previously proposed for DNA damage-responsive genes [34].

We also show that Rpd3p activity is required for normal Msn2p binding to representative promoters. This is reminiscent of the requirement of Rpd3p for nucleosome displacement and Upc2p binding at the promoters of hypoxia-regulated genes [33]. The exact mechanism of Rpd3p involvement at Msn2/4p targets is unclear; however, Lindstrom *et al*. [58] recently showed that Msn2/4p activity is inhibited by NuA4-dependent histone acetylation. This raises the possibility that histone deacetylation by Rpd3p counteracts the inhibitory effects of NuA4-dependent acetylation to allow Msn2p binding and gene induction. That different targets of Msn2/4p and Rpd3p show distinct sensitivities to the factors' deletion again implies distinct regulatory mechanisms for the different subclasses of targets. Understanding the differences in regulation will be an interesting area of future investigation.

### Rpd3p functions as a 'general-stress' co-factor in the ESR regulatory network

The ESR regulatory network consists of condition-specific regulators - those that only regulate ESR expression under specific circumstances - as well as 'general-stress' factors (such as Msn2/4p) that function under a wide variety of conditions. Our results suggest Rpd3p acts with the 'general-stress' set of ESR regulators at iESR and PS genes. Rpd3L is required for proper expression of these genes in response to numerous stresses (Figure 1). Furthermore, Msn2/4-dependent induction, but not condition-specific regulation by Hsf1p and Yap1p, requires Rpd3p (Figures 4 and 5). Like Msn2/4p, the 'general stress' role of Rpd3p persists despite the involvement of different upstream regulators under different conditions. For example, De Nadal *et al*. [32] showed that Rpd3p is recruited to numerous iESR promoters in a manner dependent on the Hog1p kinase following salt stress but independent of Hog1p after heat shock. Thus, the involvement of Rpd3p, and the transcription factors it interacts with at these promoters, is controlled by different upstream signaling pathways under different environments. It will be interesting to decipher the mechanisms by which Rpd3p associates with stress-activated transcription factors despite distinct, condition-specific upstream pathways.

### Rpd3p is required for the transient phase of stress-activated gene expression changes

This study also demonstrates the importance of histone modification in mediating rapid and transient responses to environmental changes. The Rpd3L complex is particularly important in producing the large, rapid expression changes during the period of stress acclimation. The transient expression changes produced by acute stress treatment are qualitatively distinct from continuous expression changes seen under different nutrients. However, Rpd3p can affect the rapid kinetics of both types of expression responses. Upon phosphate limitation, cells lacking *RPD3 *showed delayed induction of *PHO5 *but eventually altered expression similar to wild-type cells [59]. Interestingly, a similar effect was reported in cells lacking the histone acetyltransferase Gcn5p, which also showed delayed induction of metabolic genes [60]. These results reflect that changes in chromatin states, mediated by both deacetylases and acetyltransferases, are particularly important for rapid kinetics of gene-expression changes in response to variable environments. Consistently, we found that *rpd3Δ *cells display defects in reciprocal expression changes of the same genes upon stress exposure as well as stress relief. Dynamic and successive alterations in histone modification are crucial in producing proper transcriptional changes (for example, [61-67]). Elucidating the dynamics of chromatin changes upon stress treatment will continue to shed light on the dynamics of stress-dependent gene expression changes.

## Conclusions

Rpd3p is an important co-factor in the regulatory network that controls ESR gene expression in response to stress, working with different factors at different subsets of ESR genes. Many questions remain about the mechanistic details of Rpd3p action at these promoters. While future studies will be required to dissect the precise mechanism of Rpd3p in regulating these genes, this work contributes to our understanding of the ESR regulatory network and provides an avenue for identifying additional factors that work with Rpd3p in regulating the ESR.

## Materials and methods

### Strains and growth conditions

Strains used in this study are listed in Table S2 in Additional data file 3. *PHO23 *and *RCO1 *deletion strains were purchased from Open Biosystems (Huntsville, AL, USA), and each deletion was verified by PCR. The *rpd3Δ *and *msn2Δ msn4Δ rpd3Δ *strains were constructed by homologous recombination to replace *RPD3 *with *KANMX *or *LEU2 *in BY4741 or AGY0249, respectively. Unless otherwise noted, cells were grown at 30°C in YPD medium. Although the growth rate of the *rpd3Δ *strain is approximately 1.5-fold slower than wild type, this cannot explain the observed expression defects, since the mutant phenotypes are recapitulated by the *pho23Δ *mutant, whose doubling rate is indistinguishable from wild type.

### Cell collection for microarray analysis

Cells were grown approximately three doublings to an optical density (OD_600_) of approximately 0.6 to 0.8 and a sample was collected for the unstressed control, as previously described [68]. Basal expression in *rpd3Δ *versus wild type was measured in triplicate. For heat shock time courses, cells were grown at 25°C, filtered and resuspended in 37°C YPD. Aliquots were collected at 5, 15, 30, 45, and 60 minutes (time course HS_1) or at 5, 10, 20, 30, and 60 minutes (time course HS_2) as previously described [68]. For the H_2_O_2 _experiments, peroxide was added to 0.4 mM and cells were collected at 10, 20, 30, 40, and 60 minutes (time course H_2_O_2__1) or at 30 minutes for single-time point experiments, done in triplicate. For sodium chloride (NaCl) time courses, NaCl was added to 0.75 M and cells were collected at 15, 30, 60, and 90 minutes (time course NaCl_1) or at 30, 45, and 60 minutes (time course NaCl_2). Experiments probing the catalytically inactive *rpd3 *[32] were done in SC-leucine. The catalytically inactive *rpd3 *plasmid and the histone H4KQ mutant [38] strain were generously provided by F Posas and R Morse, respectively.

Wild-type cells were also exposed to heat shock with and without exposure to 10 μM trichostatinA (Sigma-Aldrich, St Louis, MO, USA), added 15 minutes before and throughout shock. For stress relief, cells grown at 37°C were collected by centrifugation, resuspended in 25°C YPD, and collected at 5, 10, 20, and 40 minutes (time course RH_1) or 10, 40, and 60 minutes (time course RH_2).

### Microarrays and genomic analysis

Total RNA extraction, cDNA synthesis and labeling were performed as previously described [3,68], using Superscript RT III (Invitrogen, Carlsbad, CA, USA), amino-allyl dUTP (Ambion, Austin, TX, USA) and NHS-ester cyanine dyes (Flownamics, Madison, WI, USA). Microarray data are available in the NIH Gene Expression Omnibus database with the access number [GEO:GSE9108].

Microarray data were analyzed by average-linkage hierarchical clustering, using the programs Cluster and Java-Treeview [69] as previously described [1]. Genes affected in wild-type cells were defined based on triplicate single-time-point measurements [70,71] or based on time courses [72] if *q *< 0.01 or if expression was altered more than 1.5-fold in at least two time points from replicate experiments. Genes affected in deletion strains were identified similarly, except the *q*-value cutoff was relaxed to 0.05.

### Chromatin immunopreciptation and quantitative PCR

Rpd3-myc and Msn2p ChIP experiments were done as previously described [73]. Briefly, cells were grown as described above and were either untreated or exposed to 0.4 mM H_2_O_2 _for 10 minutes, or washed twice with cold phosphate-buffered saline for the cold-shock control then exposed to 1% formaldehyde for 30 minutes (Rpd3-myc) or 45 minutes (Msn2 ChIPs) at 25°C. Cells were flash frozen, resuspended, and lysed; isolated chromatin was sonicated to an average size of approximately 400 bp. Protein (2.0 mg) was incubated with 5 μl anti-c-myc (9E11, Abcam (Cambridge, MA, USA) ab-56) or 15 μl anti-Msn2 (y-300, Santa Cruz (Santa Cruz, CA, USA) sc-33631) antibody overnight at 4°C. For chromatin ChIP, cells were exposed to 0.4 mM H_2_O_2 _for 20 minutes, cross-linked as above, then digested to spheroplasts with zymolyase (Seikagaku Biosystems, Tokyo, Japan) for 60 minutes at 30°C and treated with micrococcal nuclease (Worthington Biochemical, Lakewood, NJ, USA) for 20 minutes at 37°C to isolate mononucleosomes. This sample measured total nucleosome occupancy; in addition, 1.5 mg protein was mixed with 3 μl anti-acetylated H4 (Upstate 06-866 (Millipore, Billerica, MA, USA)) to immunoprecipitate acetylated histone H4. DNA purified from each sample was amplified [74] and converted to cDNA using SuperScript III (Invitrogen). All ChIPs were done in triplicate and quantified by real-time quantitative PCR reactions, using Sybrgreen Jumpstart Taq (Sigma-Aldrich, St Louis, MO, USA) and an Applied Biosystems 7500 detector (Foster City, CA, USA). Each ChIP PCR was normalized to a control fragment between *YEL073C *and *YEL072W *on chromosome V as previously described [75]. Apparent histone acetylation levels were normalized to nucleosome occupancy at each locus to report the fraction of acetylated nucleosomes. Primers were designed to span approximately 75 bp regions within positioned nucleosomes [76] and data not shown) and were validated by amplifying genomic DNA; primer sequences are available upon request.

## Abbreviations

ChIP: chromatin immunoprecipitation; ESR: environmental stress response; iESR: induced ESR; PAC: Polymerase A and C; PS: protein synthesis; RP: ribosomal protein; RRPE: Ribosomal RNA Processing Element.

## Authors' contributions

AAO conducted microarray analysis, data analysis, and wrote the manuscript. DJH conducted microarray analysis, ChIP studies, microscopy, data analysis, and wrote the manuscript. MS conducted reciprocal heat shift time-courses (Figure S5 in Additional data file 1), and SN carried out acquired stress experiments (Figure S6 in Additional data file 1). DP and JLW assisted in experimental setup, RNA preparation, and strain construction. APG carried out data analysis and wrote the manuscript.

## Additional data files

The following additional data are available with the online version of this paper: six supplemental figures (Figures S1 to S6; Additional data file 1); Table S1, showing enrichment of functional categories and transcription factor targets in gene groups taken from clustered expression data (Additional data file 2); Table S2, listing strains used in this study (Additional data file 3).

## Supplementary Material

Additional data file 1 Figure S1: Basal expression in *rpd3Δ *cells does not account for stress-dependent expression defects. Gene expression in unstressed *rpd3Δ *versus wild type (average of triplicate experiments) and for wild-type and *rpd3Δ *cells responding to stress is shown as in Figure 1. The middle panel shows the differences between wild-type and *rpd3Δ *expression where yellow represents higher transcript abundance (that is, weaker repression) and blue indicates lower transcript abundance (that is, weaker induction) in the *rpd3Δ *mutant. The right panel shows the difference between transcript abundance in wild-type and *rpd3Δ *cells after adjusting for the basal expression differences in unstressed cells. These data show that the observed defect in ESR initiation in the *rpd3Δ *strain is not due to the possibility that basal expression of the ESR genes already reflects the 'ON' state of the program. First, genes repressed in the ESR show little discernable difference in basal expression in *rpd3Δ *versus wild-type cells. Second, although a subset of the iESR genes are subtly derepressed (approximately 1.5-fold) in untreated *rpd3Δ *cells, the mutant still displays lower absolute transcript levels relative to wild-type at the peak of the expression response (right panel). Third, the *rpd3Δ *mutant ultimately alters expression comparable to the expression changes in wild-type cells, resulting in higher absolute transcript levels for these iESR genes in acclimated *rpd3Δ *cells compared to acclimated wild-type cells, particularly in response to heat shock and H_2_O_2 _treatment (right panel and data not shown). Finally, almost half the iESR genes show no significant difference in basal expression (within 1.3-fold of wild-type and *P *> 0.01) but are still induced to lower peak levels than in wild-type cells. Thus, the defect in stress-dependent expression changes seen in the *rpd3Δ *strain is not accounted for by basal expression differences across all ESR genes, although these results suggest that Rpd3p is required to suppress some iESR genes in the absence of stress.
Figure S2: Msn2 localizes to the nucleus upon stress treatment in cells lacking Rpd3p. Msn2p localization in wild-type and *rpd3Δ *cells responding to H_2_O_2 _treatment was scored, in cells transformed with a plasmid constitutively expressing Msn2-green fluorescent (GFP) protein obtained from T Tsukiyama [6,58]. **(a) **Percent of cells with nuclear Msn2p localization at several time points after H_2_O_2 _treatment in wild-type and *rpd3Δ *cells according to the key. **(b) **Examples of cytoplasmic and nuclear Msn2-GFP before and after stress. Nuclear Msn2p is indicated with an arrow.
Figure S3: Rpd3p catalytic activity and modifiable histone H4 are required for ESR expression in response to stress. The average expression and standard deviation of genes in the PS, RP, and iESR gene groups is shown as cells responded to **(a) **25°C to 37°C heat shock or **(b) **0.4 mM H_2_O_2 _treatment. Plots represent the response of *rpd3Δ *cells harboring plasmid-borne *RPD3 *(left), the blank vector (middle), or the catalytically inactive allele *rpd3-H150:151A *(right) according to the key. **(c) **Gene expression was also measured in wild-type cells responding to 25°C to 37°C heat shock, with and without pretreatment with 10 μM trichostatin A. The average log_2 _expression change of genes in each group is plotted for treated and untreated cells. Time points with smaller expression changes in the trichostatin A-treated cells (*P *< 0.01, paired *t*-test) are indicated with an asterisk. The effect of trichostatin A is less severe than RPD3 deletion or mutation (a, b), although still statistically significant, likely due to incomplete inhibition of Rpd3p by the drug. Each plot represents the standard deviation of expression of genes in the group (not error). **(d) **Expression of the PS, RP, and iESR genes is shown for wild-type, *rpd3Δ*, *pho23Δ*, and H4KQ cells responding to 0.4 mM H_2_O_2 _treatment, as described in Figure 1. Each column shows the average of at least biological triplicates. The difference in expression between the isogenic wild-type and each mutant strain is shown in the right panel as described in Figure 1.
Figure S4: The Rpd3L subunit *PHO23 *is required for transient initiation of the ESR. Average expression of iESR, PS, and RP genes in wild-type, *rpd3Δ*, *pho23Δ*, and *rco1Δ *cells responding to 0.4 mM H_2_O_2 _treatment at 10, 20, 30, 40, and 60 minutes.
Figure S5: Rpd3p is required for suppressed ESR expression during stress relief. **(a) **Gene expression diagrams and **(b) **average expression plots are shown as described in Figure 1 for wild-type and *rpd3Δ *cells responding to 25°C to 37°C heat shock (left panels) or the reciprocal shift from 37°C to 25°C (right panels). The starting point of the recovery experiment is indicated by a dashed line and was adjusted to the acclimated expression levels seen in the heat shock experiment for clarity.
Figure S6: *PHO23 *mutant cells display a defect in acquired stress resistance. Previous work from our lab showed that the gene expression response to a single dose of stress is not required to survive that condition, but rather protects cells against future stress [3]. Here, H_2_O_2 _tolerance in wild-type and *pho23Δ *cells was measured after a 60-minute pretreatment with 0.7 M NaCl, similarly to that previously described [3]. Briefly, log-phase cells were treated with 0.7 M NaCl (or YPD for mock-treated cells) for 60 minutes, then washed once with YPD to remove salt and exposed to 23 doses of H_2_O_2 _(ranging from 0 to 8 mM) for 2 h. Cell viability at each dose was measured using live-dead staining (Molecular Probes (Invitrogen, Carlsbad, CA, USA) and a Guava flow cytometer. The plot shows the growth score (the sum of all viability scores normalized to the starting viability score in untreated cells) for wild-type and the *pho23Δ *mutant. Error bars represent the standard deviation from triplicate experiments. The *pho23Δ *strain was not sensitive to the mild dose of NaCl in this assay (*P *= 0.4) but had a significant defect in acquired tolerance to H_2_O_2 _after mild NaCl treatment (*P *= 0.029, paired *t*-test). The defect is similar to that seen for cells lacking *MSN2 *and/or *MSN4 *[3]. Click here for file

Additional data file 2 Enrichment of functional categories and transcription factor targets in gene groups taken from clustered expression data. Click here for file

Additional data file 3 Strains used in this study. Click here for file
