# Supplementary material for: The histone deacetylase Rpd3p is required for transient changes in genomic expression in response to stress
Source: Genome Biol. 2009 May 26;10(5):R57. doi: 10.1186/gb-2009-10-5-r57 (PMC2718523; doi:10.1186/gb-2009-10-5-r57)
Supplement: Additional data file 1 — Figure S1: Basal expression in rpd3Δ cells does not account for stress-dependent expression defects. Gene expression in unstressed rpd3Δ versus wild type (average of triplicate experiments) and for wild-type and rpd3Δ cells responding to stress is shown as in Figure 1. The middle panel shows the differences between wild-type and rpd3Δ expression where yellow represents higher transcript abundance (that is, weaker repression) and blue indicates lower transcript abundance (that is, weaker induction) in the rpd3Δ mutant. The right panel shows the difference between transcript abundance in wild-type and rpd3Δ cells after adjusting for the basal expression differences in unstressed cells. These data show that the observed defect in ESR initiation in the rpd3Δ strain is not due to the possibility that basal expression of the ESR genes already reflects the 'ON' state of the program. First, genes repressed in the ESR show little discernable difference in basal expression in rpd3Δ versus wild-type cells. Second, although a subset of the iESR genes are subtly derepressed (approximately 1.5-fold) in untreated rpd3Δ cells, the mutant still displays lower absolute transcript levels relative to wild-type at the peak of the expression response (right panel). Third, the rpd3Δ mutant ultimately alters expression comparable to the expression changes in wild-type cells, resulting in higher absolute transcript levels for these iESR genes in acclimated rpd3Δ cells compared to acclimated wild-type cells, particularly in response to heat shock and H2O2 treatment (right panel and data not shown). Finally, almost half the iESR genes show no significant difference in basal expression (within 1.3-fold of wild-type and P > 0.01) but are still induced to lower peak levels than in wild-type cells. Thus, the defect in stress-dependent expression changes seen in the rpd3Δ strain is not accounted for by basal expression differences across all ESR genes, although these results suggest that Rpd3p is [file gb-2009-10-5-r57-S1.pdf]

ADDITIONAL DATAFILE 1

Figure S1

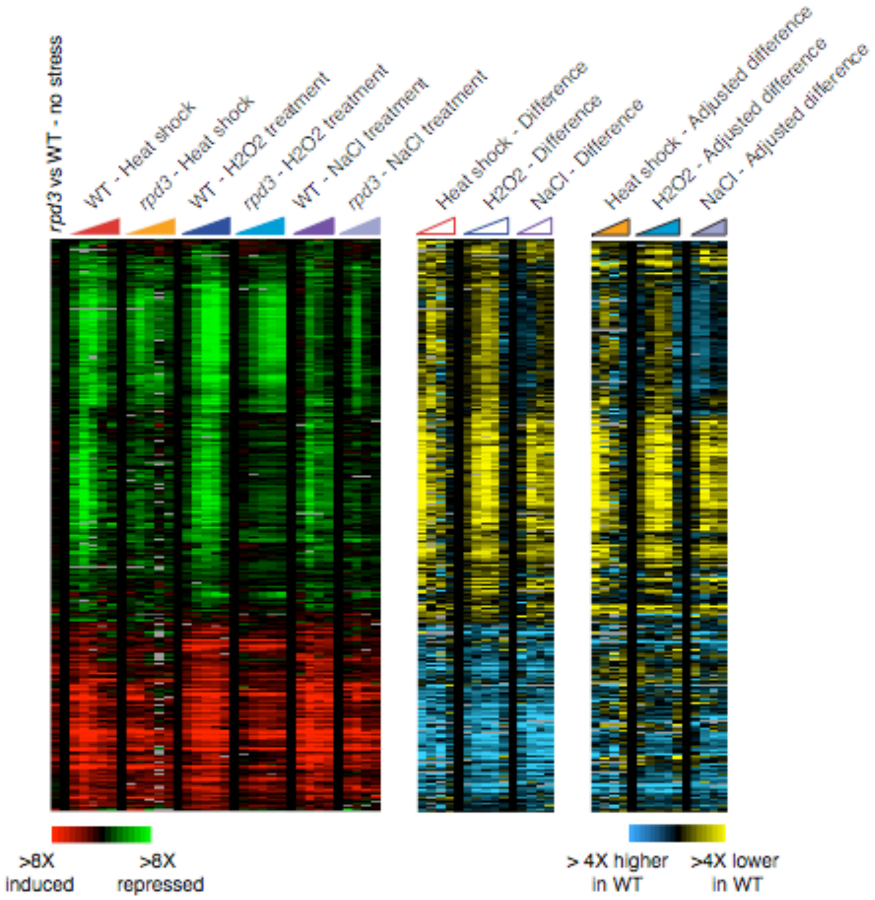

Supplemental Figure S1. Basal expression in rpd3Δ cells does not account for stress-dependent expression defects. Gene expression in unstressed *rpd3Δ* vs. wild type (average of triplicate experiments) and for wild-type and *rpd3Δ* cells responding to stress is shown as in Figure 1. The middle panel shows the differences between wild-type and *rpd3Δ* expression where yellow represents higher transcript abundance (i.e. weaker repression) and blue indicates lower transcript abundance (i.e. weaker induction) in the *rpd3Δ* mutant. The right panel shows the difference between transcript abundance in

Dana Huebert 5/21/09 5:18 PM
Formatted: Font color: Black

wild-type and *rpd3Δ* cells after adjusting for the basal expression differences in unstressed cells.

These data show that the observed defect in ESR initiation in the *rpd3Δ* strain is not due to the possibility that basal expression of the ESR genes already reflects the 'ON' state of the program. First, genes repressed in the ESR show little discernable difference in basal expression in *rpd3Δ* vs. wild-type cells. Second, although a subset of the iESR genes are subtly derepressed (~1.5X) in untreated *rpd3Δ* cells, the mutant still displays lower absolute transcript levels relative to wild-type at the peak of the expression response (right panel). Third, the *rpd3Δ* mutant ultimately alters expression comparable to the expression changes in wild-type cells, resulting in higher absolute transcript levels for these iESR genes in acclimated *rpd3Δ* cells compared to acclimated wild-type cells, particularly in response to heat shock and H_2O_2 treatment (right panel and data not shown). Finally, almost half the iESR genes show no significant difference in basal expression (within 1.3X of wild-type and $p>0.01$) but are still induced to lower peak levels than in wild-type cells. Thus, the defect in stress-dependent expression changes seen in the *rpd3Δ* strain is not accounted for by basal expression differences across all ESR genes, although these results suggest that Rpd3p is required to suppress some iESR genes in the absence of stress.

Figure S2

Dana Huebert 5/21/09 5:18 PM
Formatted: Font color: Black

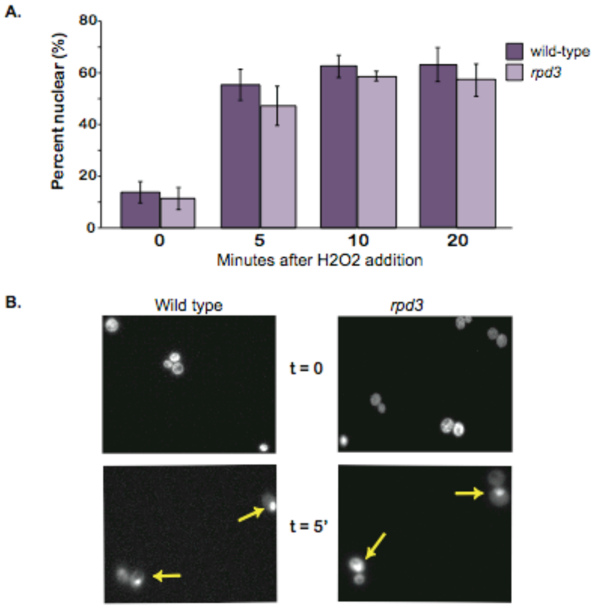

Supplemental Figure S2: Msn2 localizes to the nucleus upon stress treatment in cells lacking Rpd3p. Msn2p localization in wild-type and *rpd3*Δ cells responding to H_2O_2 treatment was scored, in cells transformed with a plasmid contitutively expressing Msn2-

GFP obtained from T. Tsukiyama [6, 58]. (A) The plot shows the percent of cells with nuclear Msn2p localization at several time points after H_2O_2 treatment in wild-type and *rpd3*Δ cells according to the key. (B) Examples of cytoplasmic and nuclear Msn2-GFP before and after stress. Nuclear Msn2p is indicated with an arrow.

Dana Huebert 5/21/09 5:18 PM
Formatted: Font color: Black

Figure S3

Dana Huebert 5/21/09 5:18 PM
Formatted: Font color: Black

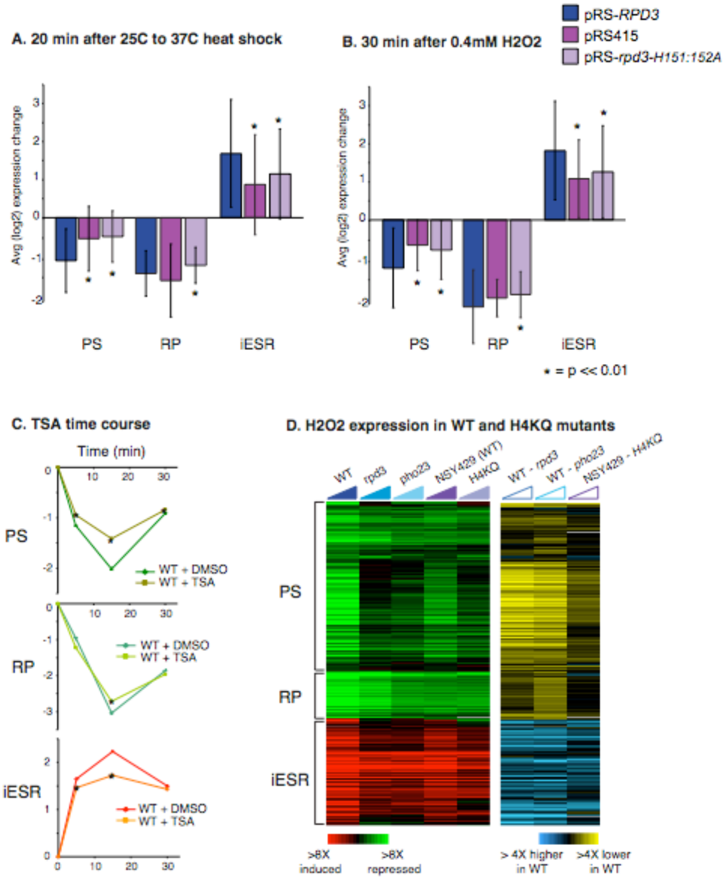

Supplemental Figure S3. Rpd3p catalytic activity and modifiable histone H4 are required for ESR expression in response to stress. The average expression and standard deviation of genes in the PS, RP, and iESR gene groups is shown as cells responded to 25°C – 37°C heat shock (A) or 0.4 mM H_2O_2 treatment (B). Plots represent the response of *rpd3Δ* cells harboring plasmid-borne *RPD3* (left), the blank vector (middle), or the catalytically inactive allele *rpd3-H150:151A* (right) according to the key. (C) Gene expression was also measured in wild-type cells responding to 25°C – 37°C heat shock, with and without pretreatment with 10 μM Trichostatin A. The average $\log_2$ expression change of genes in each group is plotted for treated and untreated cells. Time points with smaller expression changes in the TSA-treated cells ($p<0.01$, paired t-test) are indicated with an asterisk. The effect of TSA is less severe than *RPD3* deletion or mutation (A and B), although still statistically significant, likely due to incomplete inhibition of Rpd3p by the drug. Each plot represents the standard deviation of expression of genes in the group (not error). (D) Expression of the PS, RP, and iESR genes is shown for wild-type, *rpd3Δ*, *pho23Δ*, and H4KQ cells responding to 0.4 mM H_2O_2 treatment, as described in Figure 1. Each column shows the average of at least biological triplicates. The difference in expression between the isogenic wild-type and each mutant strain is shown in the right panel as described in Figure 1.

Figure S4

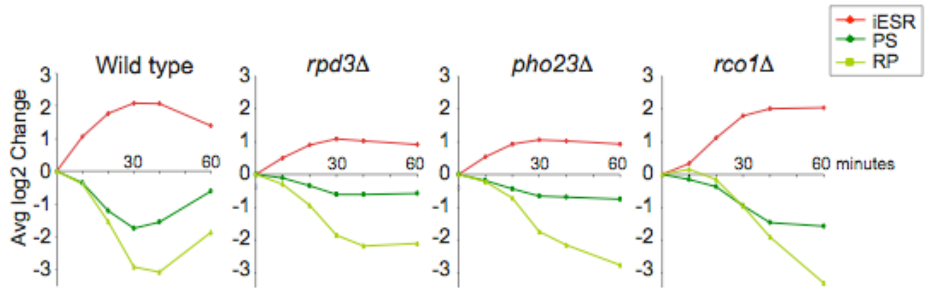

Supplemental Figure S4. The Rpd3L subunit PHO23 is required for transient initiation of the ESR. Average expression of iESR, PS, and RP genes in wild-type, *rpd3Δ*, *pho23Δ*, and *rco1Δ* cells responding to 0.4 mM H_2O_2 treatment at 10, 20, 30, 40, and 60 min after.

Dana Huebert 5/21/09 5:18 PM
Formatted: Font color: Black

Figure S5

Dana Huebert 5/21/09 5:18 PM
Formatted: Font color: Black

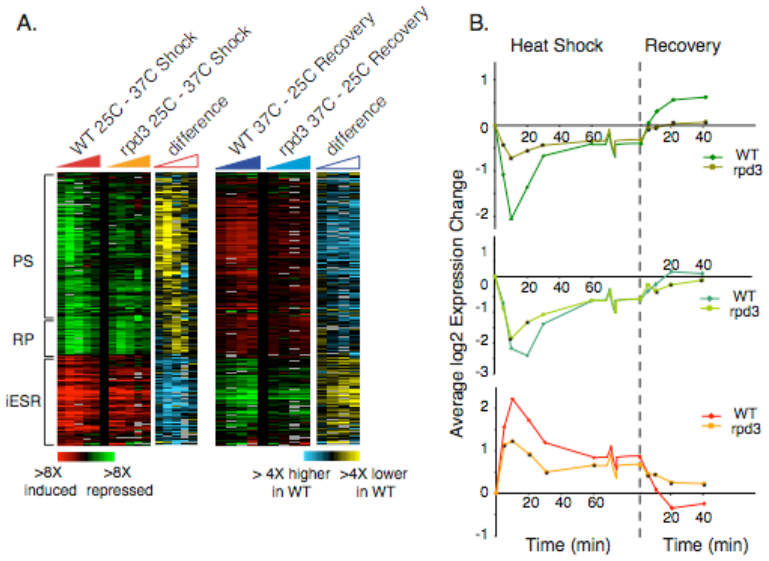

Supplemental Figure S5. Rpd3p is required for suppressed ESR expression during stress relief. Gene expression diagrams (A) and average expression plots (B) are shown as described in Figure 1 for wild-type and *rpd3Δ* cells responding to 25°C – 37°C heat shock (left panels) or the reciprocal shift from 37°C – 25°C (right panels). The starting point of the recovery experiment is indicated by a dashed line and was adjusted to the acclimated expression levels seen in the heat shock experiment for clarity.

Figure S6

Dana Huebert 5/21/09 5:18 PM
Formatted: Font:Bold, Font color: Auto

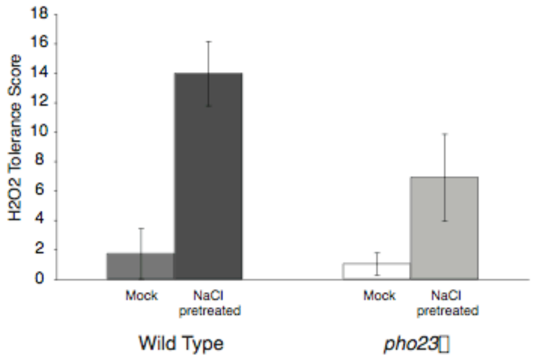

Supplemental Figure S6. Pho23 cells display a defect in acquired stress resistance.

Previous work from our lab showed that the gene expression response to a single dose of

stress is not required to survive that condition, but rather protects cells against future stress [3]. Here, H_2O_2 tolerance in wild-type and *pho23Δ* cells was measured after a 60 min pretreatment with 0.7M NaCl, similarly to that previously described [3]. Briefly, log-phase cells were treated with 0.7M NaCl (or YPD for mock-treated cells) for 60 min, then washed once with YPD to remove salt and exposed to 23 doses of H_2O_2 (ranging from 0 – 8 mM) for 2h. Cell viability at each dose was measured using live-dead staining (Molecular Probes) and a Guava flow cytometer. The plot shows the growth score (the sum of all viability scores normalized to the starting viability score in untreated cells) for wild-type and the *pho23Δ* mutant. Error bars represent the standard deviation from triplicate experiments. The *pho23Δ* strain was not sensitive to the mild dose of NaCl in this assay ($p = 0.4$) but had a significant defect in acquired tolerance to H_2O_2 after mild NaCl treatment ($p = 0.029$, paired t-test). The defect is similar to that seen for cells lacking *MSN2* and/or *MSN4* [3].

Dana Huebert 5/21/09 5:18 PM
Formatted: Font color: Black

Dana Huebert 5/21/09 5:18 PM
Formatted: Font color: Black

Dana Huebert 5/21/09 5:18 PM
Formatted: Font color: Black
